# Usefulness of ^18^F-FDG PET-CT for assessing large-vessel involvement in patients with suspected giant cell arteritis and negative temporal artery biopsy

**DOI:** 10.1186/s13075-023-03254-w

**Published:** 2024-01-04

**Authors:** Javier Narváez, Paula Estrada, Paola Vidal-Montal, Iván Sánchez-Rodríguez, Aida Sabaté-Llobera, Joan Miquel Nolla, Montserrat Cortés-Romera

**Affiliations:** 1 Department of Rheumatology, Hospital Universitario de Bellvitge - Bellvitge Biomedical Research Institute (IDIBELL), Barcelona, 08907 Spain; 2 Department of Rheumatology, Hospital de Sant Joan Despí Moisès Broggi, Barcelona, Spain; 3 Department of Nuclear Medicine – PET IDI, Hospital Universitario de Bellvitge, Barcelona, Spain

**Keywords:** Giant cell arteritis, ^18^F-FDG PET-CT, Negative temporal artery biopsy, Large-vessel involvement

## Abstract

**Objective:**

To investigate the usefulness of ^18^F-FDG PET-CT for assessing large-vessel (LV) involvement in patients with suspected giant cell arteritis (GCA) and a negative temporal artery biopsy (TAB).

**Methods:**

A retrospective review of our hospital databases was conducted to identify patients with suspected GCA and negative TAB who underwent an ^18^F-FDG PET-CT in an attempt to confirm the diagnosis. The gold standard for GCA diagnosis was clinical confirmation after a follow-up period of at least 12 months.

**Results:**

Out of the 127 patients included in the study, 73 were diagnosed with GCA after a detailed review of their medical records.

Of the 73 patients finally diagnosed with GCA, ^18^F-FDG PET-CT was considered positive in 61 cases (83.5%). Among the 54 patients without GCA, ^18^F-FDG PET-CT was considered positive in only eight cases (14.8%), which included 1 case of Erdheim-Chester disease, 3 cases of IgG4-related disease, 1 case of sarcoidosis, and 3 cases of isolated aortitis.

Overall, the diagnostic performance of ^18^F-FDG PET-CT for assessing LV involvement in patients finally diagnosed with GCA and negative TAB yielded a sensitivity of 83.5%, specificity of 85.1%, and a diagnostic accuracy of 84% with an area under the ROC curve of 0.844 (95% CI: 0.752 to 0.936). The sensitivity was 89% in occult systemic GCA and 100% in extracranial LV-GCA.

**Conclusion:**

Our study confirms the utility of ^18^F-FDG PET-CT in patients presenting with suspected GCA and a negative TAB by demonstrating the presence of LV involvement across different subsets of the disease.

## Background

Current evidence shows that giant cell arteritis (GCA) is much more than a cranial disease, as it has a much broader and heterogeneous clinical spectrum than previously thought. Generally, it exhibits a typical clinical picture consisting of classic cranial ischemic manifestations but sometimes prevail non-specific clinical features related to the general inflammatory state (occult systemic GCA presenting as fever of unknown origin and/or constitutional symptoms) or the extracranial large-vessel (LV) involvement (aorta, supra-aortic trunks, and large peripheral arteries) [1–3]. There has been an increasing knowledge of the occurrence of the disease without the typical cranial symptoms [1–6] and its close relationship and overlap with polymyalgia rheumatica (PMR), which may also be the only clinical presentation of vasculitis [7, 8]. This evidence has led to the emerging view that these diseases should be approached as linked conditions, unified under the term GCA–PMR spectrum disease (GPSD) [9].

Every effort should be made to confirm a suspected diagnosis of GCA. According to the 2018 update of the EULAR recommendations for the management of LV vasculitis, objective confirmation of the presence of vasculitis should always be obtained by imaging, with color Doppler ultrasound (CDUS) of the temporal arteries being the most commonly used imaging method, or histology (temporal artery biopsy [TAB]) [10, 11].

However, in clinical practice, it is not uncommon to encounter patients with negative cranial studies, and this subgroup remains the most challenging to diagnosis. Therefore, for patients with suspected GCA and a negative TAB or CDUS of the temporal arteries, the latest ACR guidelines recommend noninvasive vascular imaging of the large vessels in tandem with clinical assessment to aid in diagnosis versus clinical assessment alone [12]. Potential diagnostic imaging modalities include magnetic resonance imaging (MRI) or computed tomography (CT) angiography of the neck/chest/abdomen/pelvis and 18F-fluorodeoxyglucose positron emission tomography/computed tomography (^18^F-FDG PET-CT). In the 2023 update of the EULAR recommendations for the use of imaging in LV vasculitis in clinical practice [13], FDG-PET is prioritized for the detection of mural inflammation or luminal changes of extracranial arteries in patients with suspected GCA (alternatively MRI or CT).

In the present study, we review the usefulness of ^18^F-FDG PET-CT in routine clinical care as an aid to confirm the diagnosis in patients with suspected GCA and negative TAB, by demonstrating aortic and/or LV involvement.

## Methods

### Study population

The study was performed under routine clinical practice conditions. We retrospectively reviewed our hospital databases to identify all patients with suspected GCA and negative TAB who underwent an ^18^F-FDG PET-CT to try to confirm the diagnosis between January 2005 and January 2022.

After thorough examination of the medical records, two experienced rheumatologists independently validated or ruled out the diagnosis of GCA. Given the absence of formal diagnostic criteria for GCA, a reliable diagnosis was consistently established in all cases by evaluating the presence of the following six features: (1) age at disease onset ≥ 50 years; (2) the presence of compatible clinical symptoms: craniofacial ischemic symptoms (headache, scalp tenderness, abnormal temporal artery examination, jaw claudication, visual symptoms), PMR, constitutional symptoms or fever, and manifestations related to extracranial LV involvement (arm/leg claudication, pulse discrepancy, bruits of extra-cranial arteries unrelated to atherosclerosis, tenderness to palpation or decreased pulsation, inflammatory lower back pain); (3) raised acute-phase reactants (erythrocyte sedimentation rate [ESR] > 30 mm/h measured by the Westergren method or C reactive protein [CRP] > 5 mg/L); (4) objective evidence of LV vasculitis on ^18^F-FDG PET-CT (EULAR recommends confirming the presence of medium- or large-vessel vasculitis) [10, 11]; (5) prompt and persistent response to corticosteroid therapy; and (6) no change of diagnosis during a follow-up of at least 1 year.

Since our study was performed retrospectively, we were exempted by the ethics committee from obtaining informed consent. Patient information were pseudonymized prior to analysis.

### ^18^F-FDG PET/CT imaging technique and protocol

PET/CT studies were performed in a Discovery ST scanner (GE Healthcare, Milwaukee, USA), according to the specific procedural recommendation of the European Association of Nuclear Medicine (EANM), the Cardiovascular Council of the Society of Nuclear Medicine and Molecular Imaging (SNMMI), and the PET Interest Group (PIG), which was endorsed by the American Society of Nuclear Cardiology (ASNC) [14].

Patients fasted for at least 6 h and had blood glucose levels under 11 mmol/L prior to intravenous injection of FDG (185–370 MBq). Until 2020, imaging was performed 60 min after intravenous administration of the radiotracer. When it was proven that delayed image acquisition protocols beyond 60 min increased FDG-PET sensitivity when trying to detect vasculitis [15], the start of image capture was delayed to 90 min. Scans were performed in the supine position from the base of the skull to the proximal thigh; the scan region was enlarged in those case involving clinical suspicion of distal involvement. PET images were obtained at 3 min per bed position, in 3-dimensional mode, using a matrix size of 128 × 128, with a pixel size of 5.4 mm and a spatial resolution of 5.2 mm. A low-dose CT (140 kV and 80 mA) was acquired prior to the PET-emission scan for attenuation correction and anatomic localization. CT images were used for attenuation correction of the PET emission data, using the image reconstruction algorithm OSEM (ordered subset expectation maximization).

### ^18^F-FDG PET/CT imaging interpretation

Positron emission tomography/CT image scans were independently evaluated by nuclear medicine physicians with ≥ 7 years of experience in PET/CT. Each study was interpreted as active or inactive vasculitis based on an overall subjective assessment by the reader. In cases of doubt, a joint rereview of the PET/CT images was performed in a clinical session to reach a consensus.

PET images were evaluated visually and semi-quantitatively. The degree of FDG uptake in the arteries was assessed using the visual 0-to-3 vascular to liver ^18^F-FDG uptake grading scale: 0 = no uptake (≤ mediastinum); 1 = low-grade, but not negligible FDG diffuse homogeneous uptake (< liver); 2 = intermediate-grade uptake (= liver); and 3 = high-grade uptake (> liver), with grade 2 indicative of a questionable active vasculitis and grade 3 considered positive for active vasculitis [14, 16–19]. In addition to the uptake intensity, the uptake pattern was also taken into account when establishing the diagnosis of vasculitis, being indicative of wall inflammation those with a circumferential uptake and smooth linear or long segmental pattern, without wall microcalcifications [19].

For the semiquantitative analysis, automatic volumes of interest were placed and adjusted over the selected arterial region in order to obtain the maximum standardized uptake value (SUVmax).

### Statistical analysis

Results are expressed as the mean or median with standard deviation (SD), while categorical variables are presented as the number of cases and as percentages. The diagnostic performance of ^18^F-FDG PET-CT in detecting LV involvement in patients finally diagnosed with GCA and a negative TAB was assessed by calculating its sensitivity, specificity, positive likelihood ratio (LR +), negative likelihood ratio (LR-), predictive positive value (PPV), negative predictive value (NPV), accuracy, and the area under the ROC curve (AUC) with 95% confidence intervals (95% CI).

## Results

A total of 127 patients with a high clinical suspicion of GCA, negative unilateral TAB, and an ^18^F-FDG PET-CT were included. Of these patients, 40 were men and 87 were women, with a mean age of 77 ± 9 years. All subjects were initially addressed for general symptoms with a new-onset headache at some point, a girdle syndrome, or isolated persistent or severe constitutional syndrome, with raised acute phase reactants in all cases.

After a detailed review of the medical records, 73 patients were finally diagnosed as having GCA. Their main clinical features and laboratory data are summarized in Table 1. Based on their initial clinical presentation, 19 (26%) presented with isolated fever and/or constitutional syndrome (*occult systemic GCA*), 22 (30.1%) with predominantly extracranial LV involvement (*extracranial LV-GCA*), 20 (27.3%) exhibited isolated craniofacial ischemic symptoms (*isolated cranial GCA*), and 12 (16.6%) had an overlapping pattern (cranial and extracranial manifestations).

**Table 1:** Main clinical and laboratory data of the 73 patients ultimately diagnosed with GCA

Number of patients	73
Age (mean y ± SD)	76 ± 7
Women/men (ratio)	51 (70%)/22 (30%)
**Clinical features**	
Headache	44 (60.2%)
Temporal artery abnormality or scalp tenderness	26 (35.6%)
Jaw claudication	10 (13.6%)
Visual manifestations	15 (20.5%)
Cerebrovascular accidents	6 (8.2%)
Systemic symptoms^a^	60 (82.2%)
Polymyalgia rheumatica	39 (53.4%)
Limb claudication	2 (2.7%)
**Laboratory data**	
ESR (mm/h)	63 ± 27
CRP (mg/L; ref. value ≤ 5)	66.5 ± 33
Anemia (≤ 11 g/dl)	46 (63%)
Platelets (× 10^3^ cells/mm^3^)	356 ± 121
Raised ALT/AST^b^	4 (5.4%)
Raised alkaline phosphatase^b^	20 (27.3%)

Results are presented as mean ± standard deviation (SD) or number of cases with frequencies

^a^Malaise/anorexia/weight loss/fever

^b^ Increased ALT/AST and alkaline phosphatase were considered if values at diagnosis were ≥ 1.5 times normal

Of the 73 patients finally diagnosed with GCA, ^18^F-FDG PET-CT was considered positive in 61 cases (83.5%). The topography of vessel involvement in patients with positive PET-CT is shown in Table 2. The most commonly affected vessel segments were the aorta (100%), followed by the supra-aortic trunks (92%) and the large peripheral arteries (iliofemoral, axillary, and brachial arteries; 85%). Of note, PET-CT revealed shoulder, hip/greater trochanters/ischial tuberosities, or inter-spinal uptake suggestive of PMR in 39 (64%) patients.

**Table 2:** Topography of vessel involvement in patients with positive ^18^F-FDG PET-CT

	**Patients finally diagnosed with GCA** *N* = 61	**Patients without GCA** *N* = 8
**Aorta**	55 (90.1%)	4 (50%)
Thoracic aorta	55 (90.1%)	4 (50%)
*Ascending*	50 (81.9%)	3 (37.5%)
*Arch*	52 (85.2%)	4 (50%)
*Descending*	44 (72.1%)	4 (50%)
Abdominal aorta	34 (55.7%)	4 (50%)
**Supra-aortic trunks**	47 (77%)	0 (0%)
Carotid arteries	27 (44.2%)	
Subclavian arteries or brachiocephalic trunk	41 (67.2%)	
Vertebral arteries	16 (26.2%)	
**Large peripheral arteries**	31 (50.8%)	0 (0%)
Iliac arteries	21 (34.4%)	
Femoral arteries	28 (45.9%)	
Axillary arteries	19 (31.1%)	
Brachial arteries (proximal third)	6 (9.8%)	

Thirty-eight patients (52%) received glucocorticoid (GC) treatment before undergoing PET-TC (refer to Table 3): 29 (47.5%) of the patients with a positive PET-TC result and 9 (75%) with a negative scan. Among the latter group, seven out of the nine received intravenous methylprednisolone boluses (at doses of 125 mg to 1 g per day for 3 days) followed by 30 to 60 mg/day of prednisone due to severe ischemic complications (visual manifestations or stroke). The median duration of treatment prior to the PET-CT was 3 days (range, 3–13).

**Table 3:** 18F-FDG PET-CT results in patients with and without prior glucocorticoid treatment

**GCA patients (*N* = 73)**	
*Positive *^*18*^*F-FDG PET-CT (N* = *61)*	
Prior treatment with GC	29 (47.5%)
No	32 (52.5%)
*Negative *^*18*^*F-FDG PET-CT (N* = *12)*	
Prior treatment with GC	9 (75%)
No	3 (25%)
**Non GCA patients (*N* = 54)**	
*Positive *^*18*^*F-FDG PET-CT (N* = *8)*	
Prior treatment with GC	6 (75%)
No	2 (25%)
*Negative *^*18*^*F-FDG PET-CT (N* = *46)*	
Prior treatment with GC	14 (30.4%)
No	32 (69.6%)

The remaining 54 patients had other final diagnoses: other forms of vasculitis (*N* = 3), non-arteritic anterior ischemic optic neuropathy, pure PMR, cases of non-specific or tensional headache with only imaging evidence of atherosclerosis (most of these patients had chronic kidney disease), amyloidosis, Erdheim-Chester disease, IgG4-related disease, sarcoidosis, infections, and malignancies. Their main clinical features and laboratory data are summarized in Table 4.

**Table 4:** Main clinical and laboratory data of the 54 patients without GCA (“controls”)

Number of patients	54
Age (mean y ± SD)	73 ± 6
Women/men (ratio)	35 (66%)/19 (34%)
**Clinical features**	
Headache	41 (76%)
Temporal artery abnormality or scalp tenderness	5 (9%)
Visual symptoms	8 (15%)
Systemic symptoms^a^	46 (85%)
Girdle syndrome	24 (44%)
Vascular bruit	2 (3.7%)
**Laboratory data**	
ESR (mm/h)	59 ± 26
CRP (mg/L; ref. value ≤ 5)	26 ± 15
Anemia (≤ 11 g/dl)	46 (63%)
Platelets (× 10^3^ cells/mm^3^)	331 ± 149
Raised ALT/AST^b^	7 (13%)
Raised alkaline phosphatase^b^	6 (11%)

Results are presented as mean ± standard deviation (SD) or number of cases with frequencies

^a^Malaise/anorexia/weight loss/fever

^b^Increased ALT/AST and alkaline phosphatase were considered if values at diagnosis were ≥ 1.5 times normal

Of the 54 patients without GCA, ^18^F-FDG PET-CT was considered positive in only 8 (14.8%) patients: 1 case of Erdheim-Chester disease, 3 IgG4-related diseases, 1 case of sarcoidosis, and 3 cases with Isolated aortitis [20]. In these eight patients, only the aorta was affected (see Table 2).

Twenty of these 54 patients (37%) had previously been treated with GC at 30 to 60 mg/day of prednisone (see Table 3): 6 (75%) of the patients with a positive PET-TC result and 14 (30.4%) with a negative scan. The median duration of treatment before the PET-TC was 4 days (range, 2–16).

### Diagnostic performance of ^18^F-FDG PET-CT for assessing large-vessel involvement in patients finally diagnosed with GCA

Overall, PET-CT had a sensitivity of 83.5% (95% CI: 73% to 91.2%), a specificity of 85.1% (72.8% to 93.3%), a LR + of 5.6 (2.95 to 10.78), a LR − of 0.19 (0.11 to 0.33), a PPV of 88.3% (79.9% to 93.5%), a NPV of 79.3% (69.3% to 86.7%), and a diagnostic accuracy of 84% (76.7% to 90.1%) with an AUC of 0.844 (0.752 to 0.936).

According to the clinical phenotypes at presentation, the sensitivity was 89% (66.8% to 98.7%) in occult systemic GCA, 100% (84.5% to 100%) in extracranial LV-GCA, and 68.7% (49.9% to 83.8%) in patients with isolated cranial GCA or with an overlapping pattern (cranial and extracranial) with negative TAB.

### Diagnostic performance of ^18^F-FDG PET-CT in assessing large-vessel involvement

When evaluating the utility of ^18^F-FDG PET-CT in detecting LV involvement across the entire sample, encompassing patients with a final diagnosis of GCA and those with other conditions associated with aortitis (Erdheim-Chester disease, IgG4-related diseases, sarcoidosis, and isolated aortitis), the PET-CT demonstrated a sensitivity of 85.2% (95% CI: 75.5% to 92.1%), a specificity of 100% (92.3% to 100%), a LR − of 0.15 (0.09 to 0.25), a PPV of 100%, a NPV of 83.3% (74.8% to 89.4%), and an overall diagnostic accuracy of 91.5% (85.2% to 95.7%).

## Discussion

Diagnosing GCA remains challenging in patients with a negative TAB, particularly in those with exclusively LV involvement. Many factors may affect TAB yield, such as the biopsy length, the duration of previous corticosteroid therapy, or the absence of temporal artery involvement in GCA [21, 22]. In this sense, current evidence shows that GCA is much more than a cranial disease, as it has a much broader and heterogeneous clinical spectrum than previously thought [1–9].

Generally, it typically presents with classic cranial ischemic manifestations. However, non-specific clinical features related to the general inflammatory state (occult systemic GCA) or the extracranial LV involvement can also occur, without the involvement of the external carotid arterial branches. Patients with extracranial LV-GCA often present with a girdle syndrome, which usually occurs at an earlier age and can be associated with constitutional symptoms, as well as atypical symptoms such as inflammatory lower back pain or claudication of the upper and lower limb [3–9]. These patients have an increased risk of aortic complications (such as aortic aneurysm and dissection, aortic arch syndrome, and limb arterial stenosis) and/or cardiovascular events, and a greater risk of relapse during the follow-up [4–8].

Although the advent of new imaging techniques has proven to help identify patients with occult systemic GCA or extracranial LV-GCA (including those with refractory or atypical PMR) without the classic cranial manifestations of the disease, there is no uniform consensus on the best imaging techniques to use, which depends on the experience and availability of each center [11].

According to our study, ^18^F-FDG PET-CT is a useful imaging technique for diagnosing patients with suspected GCA and a negative TAB. It identified LV involvement in most GCA patients across different disease subsets and helped in therapeutic decision-making. The overall sensitivity for the total sample was 83.5%, with varying sensitivity for different clinical phenotypes: 89% in occult systemic GCA, 100% in extracranial LV-GCA, and 68.7% in patients with isolated cranial GCA or with an overlapping pattern (cranial and extracranial).

Its sensitivity in real-life clinical practice is in accordance with data reported in 3 meta-analyses that analyzed the diagnostic accuracy of ^18^F-FDG PET-CT for LV vasculitis (sensitivity ranged from 80 to 90%) [23–25]. In another retrospective study of 63 patients with suspected GCA and negative TAB, ^18^F-FDG PET-CT showed LV involvement in 22 patients, 14 of whom were finally diagnosed with GCA; overall, ^18^F-FDG uptake by LV yielded a sensitivity of 61% and a specificity of 80% [26]. In this study, many patients were treated with corticosteroids before PET-CT.

^18^F-FDG PET-CT can also distinguish vasculitis from atherosclerotic lesions and detect inflammation of the periarticular and extra-articular synovial structures in PMR. In addition, from our point of view, this imaging technique offers the advantage of ruling out other diseases that can also present with headache and/or girdle and constitutional syndrome mimicking GCA, such as infections, malignancies, or other systemic inflammatory diseases.

Although ^18^F-FDG PET-CT is one of the most sensitive exams to detect vascular involvement in GCA, this imaging modality is still not readily available in many centers. The inclusion of FDG-PET in the new 2022 ACR/EULAR classification criteria [27] may potentially increase its relevance to GCA, ultimately facilitating future widespread access to this test. However, further steps should be taken to define which patients with suspected GCA would benefit from undergoing PET-CT or other imaging modalities. Thus, our study confirms its usefulness when the diagnosis is uncertain following a negative TAB across all clinical phenotypes, including those patients with non-classic disease presentations, such as extracranial LV-GCA or occult systemic GCA.

When interpreting the study results, several potential limitations must be considered. These encompass (1) the study’s observational and retrospective design; (2) the relatively small sample size; (3) potential selection bias, as the indication for PET was likely influenced by specific clinical features indicative of a high pre-test probability for the condition; (4) the challenge of “circular testing,” where the investigated imaging method was part of the diagnostic criteria for GCA; and (5) the awareness of PET results by physicians confirming the diagnosis. All of these factors may inflate the diagnostic properties of PET-CT.

However, it is important to note that there is currently no gold standard for non-cranial GCA diagnosis, as obtaining an aortic or large-artery biopsy is not feasible unless surgery is required. Therefore, testing 18F-FDG PET-CT against a pragmatic reference diagnosis becomes crucial. Our study specifically addresses a common scenario encountered in clinical practice: patients with suspected GCA and negative cranial studies, assessing the utility of PET-CT for obtaining objective confirmation of the presence of medium- or large-vessel vasculitis. It is important to recognize that our results may not be generalizable to other clinical scenarios with a lower pre-test probability.

The data presented in this study reflect outcomes observed in realistic clinical practice settings. In all instances, the conclusive diagnosis of GCA was established by considering both typical clinical symptoms and the confirmation of medium- or large-vessel vasculitis through imaging. The approach employed aligns with recommendations from EULAR on imaging [11] and management [10] of LV vasculitis, as well as the most recent guidelines from ACR [12], advocating for the application of this integrated diagnostic strategy.

An additional limitation involves the prior administration of glucocorticoids (GC) before ^18^F-FDG PET-CT in 45.6% (58/127) of patients. GC treatment may diminish the vascular wall uptake of ^18^F-FDG while increasing FDG uptake in the liver, potentially leading to underestimated vasculitis [14]. To mitigate this effect, it is recommended that ^18^F-FDG PET-CT be conducted promptly, ideally within the initial 3 to 10 days following the initiation of high-dose steroid treatment. When this is not feasible, a recent study conducted under routine clinical practice conditions demonstrates that its diagnostic yield can be valuable in a non-negligible percentage of patients with new onset GCA within the first 6 weeks of treatment, except when IV boluses are administered at MP doses > 125 mg [19].

Supporting this observation, a prospective study revealed that PET-CT identified LV disease in the majority (71.4%) of GCA patients, even after the initiation of glucocorticoid therapy, with comparable uptake [28]. Similarly, in the previously mentioned study by Hay et al. [26], corticosteroid therapy did not significantly affect diagnostic performance, although there was a trend toward lower sensitivity in patients receiving corticosteroid therapy for more than 3 days.

## Conclusions

In summary, our study confirms the usefulness of 18F-FDG PET-CT in patients with suspected GCA and negative TAB, by demonstrating the presence of LV involvement across different subsets of the disease under routine care. This study is the largest conducted to date that explicitly addresses this question.

However, it is important to note that our results should not be extrapolated to other clinical scenarios with a lower pre-test probability. Further investigations in other populations are needed to validate our findings.

## Data Availability

All relevant data generated or analyzed during this study are included in this published article and supplementary information files.

## Abbreviations

ASNC: American Society of Nuclear Cardiology
AUC: Area under the ROC curve
CDUS: Color Doppler ultrasound
CI: Confidence interval
CRP: C reactive protein
CT: Computed tomography
^18^F-FDG PET-CT: 18F-Fluorodeoxyglucose positron emission tomography/computed tomography
EANM: European Association of Nuclear Medicine
ESR: Erythrocyte sedimentation rate
GCA: Giant cell arteritis
GPSD: GCA–PMR spectrum disease
LR +: Positive likelihood ratio
LR-: Negative likelihood ratio
LV: Large vessel
MRI: Magnetic resonance imaging
NVR: Negative predictive value
PIG: PET Interest Group
PMR: Polymyalgia rheumatica
PPV: Predictive positive value
SD: Standard deviation
SNMMI: Society of Nuclear Medicine and Molecular Imaging
SUVmax: Maximum standardized uptake value
TAB: Temporal artery biopsy
